# Psychometric re-evaluation of the German version of the Physicians’ Reaction to Uncertainty Scale

**DOI:** 10.3389/fpsyg.2025.1552177

**Published:** 2025-08-06

**Authors:** David Zybarth, Laura Inhestern, Corinna Bergelt

**Affiliations:** ^1^Department of Medical Psychology, University Medical Center Hamburg-Eppendorf, Hamburg, Germany; ^2^Department of Medical Psychology, Greifswald University Hospital, Greifswald, Germany

**Keywords:** uncertainty, physicians, psychometrics, surveys and questionnaires, Physicians’ Reaction to Uncertainty Scale

## Abstract

**Introduction:**

Uncertainties are an integral part of medicine and can lead to adverse effects if not addressed. However, assessing these uncertainties can be a challenging task. The Physicians’ Reaction to Uncertainty (PRU) scale is a widely used tool to assess behavioral and emotional reactions to uncertainty. This study aims to examine the factorial structure and re-evaluate the psychometric properties of the German version in a heterogeneous sample of physicians.

**Methods:**

We conducted an online survey among German physicians, irrespective of their medical specialties. We calculated means, standard deviations, and difficulty and discrimination indices for the items. We computed Cronbach’s alpha for all subscales. We used confirmatory factor analyses to assess factorial validity due to discrepancies in the assignment of item 5 between the original and the German versions. In the original version, item 5 was assigned to the subscale “anxiety due to uncertainty.” In contrast, in the, based on exploratory factor analysis, it was assigned to the subscale “concern about bad outcomes.” We tested three models: Model 1 (the original version), Model 2 (the German version), and Model 3 (which excludes item 5).

**Results:**

A total of 136 physicians (59% were women) from 22 different medical specialties participated in this survey. The German version exhibited good psychometric properties. Most item difficulties were acceptable, and all items demonstrated good item discrimination indices. The Cronbach’s alpha values were satisfactory for all subscales. Confirmatory factor analyses revealed a Heywood case for all models, which necessitated setting the variance of item 14 to zero. The fit indices for Models 1 and 2 were insufficient. Model 3 showed superior fit indices (robust root mean square error of approximation = 0.064, robust Tucker–Lewis index = 0.952, robust comparative fit index = 0.962). The Akaike’s information criterion and Bayesian information criterion statistics indicated a substantially better fit for Model 3 compared to Models 1 and 2.

**Discussion:**

The 14-item German version of the Physicians’ Reaction to Uncertainty Scale demonstrated good psychometric properties in a heterogeneous sample of physicians. It serves as a valuable tool for assessing uncertainty and facilitating international comparisons of uncertainty scores and training effects. However, the German version appears to require further adaptation, particularly regarding item 5.

## Introduction

Life, and therefore medicine, is inherently characterized by uncertainties. When a patient seeks medical aid for a common cold, it is highly likely that the cause of the symptoms is benign. Nevertheless, there are instances where flu-like symptoms may indicate a serious disease, as described by Chu and Yip (2019). Distinguishing between the two scenarios can be challenging and might cause stress for physicians who struggle with tolerating uncertainty. According to Han et al. (2011), uncertainty is defined as metacognitive awareness of ignorance. In a narrative review, Hillen et al. (2017) propose a multidimensional conceptual model of uncertainty tolerance. Their conceptualization of uncertainty tolerance expands Han’s definition by incorporating both negative and positive psychological responses elicited by uncertainty. These psychological responses can be categorized into three domains: cognitive, affective, and behavioral. The specific positive or negative response (e.g., interpreting a situation as a threat vs. interpreting it as an opportunity) depends on various factors, including individual characteristics, situational contexts, and cultural influences (Hillen et al., 2017). Advancements in medicine, such as the rise of artificial intelligence, introduce new uncertainties, including model-driven and data-driven uncertainties (Seoni et al., 2023). Thus, considering a physician’s tolerance for uncertainty is of critical importance. A lower level of uncertainty tolerance is correlated with reduced job satisfaction and increased burnout rates (Hancock and Mattick, 2020). Additionally, it is associated with specific behaviors, such as unnecessary referrals and an over- or misuse of diagnostic tests (Scott et al., 2023). These behaviors contribute to higher costs for the healthcare system in general and might strain the patient–physician relationship (Begin et al., 2022). Given the negative consequences associated with low uncertainty tolerance, there is a need for psychometrically validated instruments to assess this construct accurately.

To evaluate an individual’s tolerance for uncertainty, several instruments exist [e.g., the Intolerance of Uncertainty Scale by Carleton et al. (2007)]. One of the most established instruments specifically for physicians is the Physicians’ Reaction to Uncertainty (PRU) scale (Gerrity et al., 1995). The scale measures affective and behavioral reactions to uncertainty. It comprises four subscales: “anxiety due to uncertainty,” “concern about bad outcomes,” “reluctance to disclose uncertainty to patients,” and “reluctance to disclose mistakes to physicians.” Depending on the specific research question, a stand-alone use of a single subscale is appropriate, which is a notable benefit of the PRU scale compared to other instruments (Stephens et al., 2023). The scale was translated, culturally adapted for the German context, and validated using a sample of 93 general practitioners (Schneider et al., 2007). The authors acknowledged that the homogeneity of their sample might have biased the results. Furthermore, this limitation restricts the generalizability of the German version of the PRU scale. Given that the medical specialty is significantly associated with levels of uncertainty (Bovier and Perneger, 2007), this lack of variety raises particular concern. Conducting a psychometric re-evaluation with a larger and more heterogeneous sample of physicians from various medical specialties would enhance the representativeness and external validity of the PRU scale. Additionally, the originally proposed factor structure was not fully confirmed, and the factorial assignment of item 5 was altered (Schneider et al., 2007). In the German version, based on an exploratory factor analysis, the item was attributed to the subscale “concern about bad outcomes.” However, there was a substantial factor loading of the item for the original subscale “anxiety due to uncertainty.” A re-evaluation could assess the stability of the modified factor structure.

To further assess the quality of the German version of the PRU scale, this study aims to (a) evaluate the psychometric properties of the PRU scale that include difficulty and discrimination indices and internal consistency, in a heterogeneous group of physicians and (b) examine the factorial structure as proposed by Schneider et al. (2007) through confirmatory factor analysis.

## Materials and methods

### Study design and recruitment

We conducted a cross-sectional online survey that included several questionnaires, as well as basic sociodemographic and job-related variables. The survey targeted physicians, irrespective of their medical specialties. Participants were allowed to select multiple specialties rather than being restricted to just one. No further inclusion or exclusion criteria were applied. The study received approval from the Local Psychosocial Ethics Committee of the Center for Psychosocial Medicine at the Medical Center Hamburg-Eppendorf (LPEK-0372).

The German version of the Physicians’ Reaction to Uncertainty Scale (PRU) (Schneider et al., 2007) was part of the above-mentioned online survey, which was administered using LimeSurvey (version 2.62.2 + 170,203) between January 2022 and December 2022. To recruit physicians from different medical specialties, we used multiple recruitment strategies. We contacted the medical chambers of each German federal state, professional societies (Hartmannbund – Verband der Ärztinnen und Ärzte Deutschlands e.V., Marburger Bund, Deutscher Hausärzteverband e.V., Berufsverband der Kinder- und Jugendärzt*innen e.V., Deutsche Gesellschaft für Kinder- und Jugendmedizin e.V.) and used newsletters and mailing lists of different organizations (Hamburger Netzwerk für Versorgungsforschung, KEKS e.V., Department of General Practice and Primary Care at Medical Center Hamburg-Eppendorf) as well as social media posts. Prior to participation, all physicians were required to provide informed consent. For confirmatory factor analyses, we targeted a minimum sample size of 200 participants (Jackson et al., 2013).

### Sample characteristics

Despite prolonged efforts utilizing the mentioned strategies, we were unable to recruit a minimum of 200 survey participants. A total of 203 physicians initiated the survey by clicking on the link at least once. Of these, 66 participants did not complete the questionnaire or did not participate in the survey at all. Out of the remaining 137 participants, one person was excluded from the analyses as an outlier, after selecting only the first answer option. This resulted in a sample of 136 participants. We assessed demographic and job-related variables, such as gender, age (classified into categories to ensure anonymity), and medical specialty. Approximately 59% of the participants were women. The participants reported 22 different medical specialty backgrounds (e.g., ophthalmology, (neuro)surgery, gynecology, and neurology; see Supplementary File 1 for a comprehensive list), with the majority specializing in “internal and general medicine” (*n* = 45) and “pediatrics and adolescence medicine” (*n* = 42). Detailed information can be found in Table 1. A chi-squared test was conducted to examine the independence between gender and medical specialty. The results indicated no significant association between these variables (χ^2^(2) = 1.71, *p* = 0.43). A second chi-squared test was performed to assess the independence between age group and medical specialty, which also yielded a non-significant result (χ^2^(6) = 5.56, *p* = 0.48).

**Table 1:** Sociodemographic characteristics of participating physicians (*n* = 136).

Sample characteristic	*n*	%
Gender		
Men	56	41
Women	80	59
Age		
30–40	38	28
41–50	40	29
51–60	29	21
>60	21	15
Specialty**		
General and internal medicine	45	33
Pediatrics and adolescent medicine (other)	42	31
General and internal medicine	64	47

** Multiple answers possible.

### Instruments

The Physicians’ Reaction to Uncertainty Scale comprises 15 items, which are assigned to four subscales (Table 2). Participants respond using a 6-point Likert scale, ranging from 1 (“Strongly disagree”) to 6 (“Strongly agree”). The subscale scores are computed by summing the item scores, with higher scores indicating increased difficulty. Items 4, 9, 10, and 12 are inversely coded. Notably, in the German version, which is available in the Supplementary File 2, the factorial assignment of item 5 (“The uncertainty of patient care often troubles me.”), based on principal component analysis, was altered from the subscale “anxiety due to uncertainty” to “concern about bad outcomes.” Despite this change, item 5 still demonstrated a high factor loading on the anxiety subscale (0.44).

**Table 2:** Physicians’ Reaction to Uncertainty (PRU) Scale.

Subscale	Item
Anxiety due to uncertainty	1) I usually feel anxious when I am not sure of a diagnosis.
	2) I find the uncertainty involved in patient care disconcerting.
	3) Uncertainty in patient care makes me uneasy.
	4) I am quite comfortable with the uncertainty in patient care.*
	5) The uncertainty of patient care often troubles me.
Concern about bad outcomes	6) When I am uncertain of a diagnosis, I imagine all sorts of bad scenarios—patient dies, patient sues, etc.
	7) I fear being held accountable for the limits of my knowledge.
	8) I worry about malpractice when I do not know a patient’s diagnosis.
Reluctance to disclose uncertainty to patients	9) When physicians are uncertain of a diagnosis, they should share this information with their patients.*
	10) I always share my uncertainty with my patients.*
	11) If I shared all my uncertainties with my patients, they would lose confidence in me.
	12) Sharing my uncertainty improves my relationship with my patients.*
	13) I prefer patients not to know when I am uncertain of what treatments to use.
Reluctance to disclose mistakes to physicians	14) I almost never tell other physicians about diagnoses I have missed.
	15) I never tell other physicians about patient care mistakes I have made.

Rating of items on a 6-point Likert scale ranging from “strongly disagree” to “strongly agree”; * Items are inversely coded.

### Data analysis

Analysis was conducted using R version 4.3.2 (R Core Team, 2023), along with the MVN, an R package (Korkmaz et al., 2014), cocron (Diedenhofen and Musch, 2016), and lavaan packages (Rosseel, 2012). The analyses and reporting followed the recommendations of Streiner and Kottner (2014). Participants were not allowed to skip questions. Therefore, only complete questionnaires were included in the analyses, and no questionnaire data were missing. To describe sample and item/scale characteristics, we reported frequencies for categorical variables, as well as means and standard deviations for items and scales.

To improve comparability, our analyses were aligned with the methodology outlined in Schneider et al. (2007). Item characteristics for each item of the PRU scale were evaluated using difficulty and discrimination indices. Recommended values for acceptable item difficulty indices range from 0.2 to 0.8, while values above 0.5 are considered indicative of good item discrimination (Döring and Bortz, 2016). Cronbach’s alpha was used to assess the internal consistency of the subscales, interpreting values greater than 0.7 as satisfactory (Bland and Altman, 1997; Cronbach, 1951). Factorial validity was examined through confirmatory factor analyses utilizing robust maximum likelihood estimators.

In light of the differences between the German and English versions (Gerrity et al., 1995; Schneider et al., 2007), we tested three models:

1. Model 1 corresponds to the original English version (Gerrity et al., 1995), with item 5 assigned to the subscale “anxiety due to uncertainty.”
2. Model 2 corresponds to the German version (Schneider et al., 2007), with item 5 assigned to the subscale “concern about bad outcomes.”
3. Model 3 excludes item 5 due to the ambiguous wording of the German translation.

The assumption of multivariate normality was assessed using Mardia’s test (Mardia, 1974). Potential violations of multivariate normality were corrected using a Satorra-Bentler scaled test statistic. To evaluate the model fit, we used the following standard indices: CMIN/df statistics, the root mean square error of approximation (RMSEA), standardized root mean square residual (SRMR), the Tucker–Lewis index (TLI), and the comparative fit index (CFI). For CMIN/DF, values ≤ 3 indicate an acceptable fit (Kline, 1998). An RMSEA score of < 0.06 is considered acceptable, as well as an SRMR score of <0.08, while TLI and CFI values >0.95 are deemed favorable (Hu and Bentler, 1999). To assess differences between models, we utilized Akaike’s information criterion (AIC) (Akaike, 1974) and the Bayesian information criterion (BIC) (Schwarz, 1978). Smaller AIC and BIC values indicate better overall model fit, with differences greater than *Δ* 2 interpreted as meaningful (Burnham and Anderson, 2002; Raftery, 1995). The discriminant validity of highly correlated factors is assessed in line with the guidelines provided by Rönkkö and Cho (2022). They propose a classification based on two criteria: (1) the upper limit of the 95% confidence interval of the factor correlation and (2) the magnitude of the factor correlation in conjunction with the difference between the chi-squared test values of the original model and a more constrained model.

## Results

### Item and scale characteristics and reliability

Tables 3, 4 provide an overview of the item and scale characteristics along with their corresponding reliability scores. The item difficulty index ranged from 0.18 for item 14 to 0.56 for item 1. Besides item 14, the remaining items showed acceptable difficulty scores. All subscales demonstrated Cronbach’s alpha values within the defined range, with a minimum value of 0.8, regardless of whether item 5 was assigned to the subscale “anxiety due to uncertainty” or to the subscale “concern about bad outcomes.” Cronbach’s Alpha did not significantly differ from the results reported by Gerrity et al. (1995) for the subscales “anxiety due to uncertainty” (χ^2^(1) = 0.16, *p* = 0.69) and “reluctance to disclose mistakes to physicians” (χ^2^(1) = 3.2, *p* = 0.07). The values for the remaining subscales were significantly higher in our sample: “concern about bad outcomes”: χ^2^(1) = 17.33, *p* < 0.01, and “reluctance to disclose uncertainty to patients”: χ^2^(1) = 11.3, *p* < 0.01. Since Gerrity et al. (1995) did not explicitly report the number of participants for each subscale (participants ranged from *n* = 257 to *n* = 262 for subscales), we used the lower bound of *n* = 257 for all comparisons of Cronbach’s Alpha.

**Table 3:** Item characteristics based on *n* = 136 physicians.

Variable	M	SD	P_i_	γ_1_	γ_2_
Item 1	3.8	1.5	0.56	−0.22	−1.02
Item 2	3.2	1.5	0.44	0.2	−1.02
Item 3	3.6	1.5	0.51	−0.2	−0.92
Item 4	2.9	1.4	0.39	0.36	−0.71
Item 5	3.2	1.4	0.43	0.08	−0.87
Item 6	2.7	1.5	0.34	0.48	−0.88
Item 7	3.1	1.5	0.43	0.28	−0.9
Item 8	3	1.6	0.39	0.31	−1.01
Item 9	2	1.1	0.19	1.26	1.43
Item 10	2.8	1.3	0.36	0.64	−0.27
Item 11	2.8	1.6	0.36	0.56	−0.86
Item 12	2.5	1.3	0.29	0.83	0.34
Item 13	2.4	1.2	0.27	0.5	−0.78
Item 14	1.9	1.1	0.18	1.67	2.82
Item 15	2	1.3	0.2	1.52	1.72

M = mean, SD = standard deviation, P_i_ = item difficulty index, γ_1_ = skewness, γ_2_ = kurtosis.

**Table 4:** Scale characteristics and Cronbach’s alpha based on *n* = 136 physicians.

Scale	Anxiety due to uncertainty	Concern about bad outcomes	Reluctance to disclose uncertainty to patients	Reluctance to disclose mistakes to physicians
M (SD)^a^	16.6 (5.9)	8.8 (4.1)	12.3 (5.5)	3.9 (2.2)
Alpha^a^	0.87	0.88	0.89	0.8
M (SD)^b^	13.5 (4.9)	12 (5.1)	12.3 (5.5)	3.9 (2.2)
Alpha^b^	0.87	0.87	0.89	0.8
M (SD)^c^	13.5 (4.9)	8.8 (4.1)	12.3 (5.5)	3.9 (2.2)
Alpha^c^	0.87	0.88	0.89	0.8
M (SD)^d^	17.6 (6.2)	8.2 (3.9)	14.9 (5.2)	4.1 (2.2)
Alpha^d^	0.87	0.87	0.86	0.91
M (SD)^e^	18.8 (4.7)	9.5 (3.1)	13.6 (4.2)	4.4 (1.9)
Alpha^e^	0.86	0.73	0.79	0.72

M (SD) = mean (standard deviation); Alpha = Cronbach’s alpha.

a Item 5 assigned to subscale “anxiety due to uncertainty.”

b Item 5 assigned to subscale “concern about bad outcomes.”

c Questionnaire without item 5.

d Results of Schneider et al. (2007).

e Results of Gerrity et al. (1995).

To examine differences in subscale means between our sample and the results reported by Gerrity et al. (1995), Welch’s *t*-tests were conducted. These revealed significant differences across most subscales, except for “concern about bad outcomes” (*t*(218.49) = 1.74, *p* = 0.08). Significant differences were observed for “anxiety due to uncertainty” (*t*(227.37) = 3.76, *p* < 0.01), “reluctance to disclose mistakes to physicians” (*t*(220.12) = 2.41, *p* < 0.05), and “reluctance to disclose mistakes to physicians” (*t*(242.66) = 2.24, *p* < 0.05). Subscale means between our study and the results reported by Schneider et al. (2007) revealed no significant differences for “anxiety due to uncertainty” (*t*(191.24) = 1.22, *p* = 0.22), “concern about bad outcomes” (*t*(204.15) = −1.12, *p* = 0.26), and “reluctance to disclose mistakes to physicians” (*t*(197.81) = 0.68, *p* = 0.5). Only the means of the subscale “reluctance to disclose uncertainty to patients” showed a significant difference (*t*(204.89) = 3.63, *p* < 0.01). The item discrimination indices (Table 5) ranged from 0.59 to 0.83, depending on the assignment of item 5 to either the subscale “anxiety due to uncertainty,” the subscale “concern about bad outcomes,” or the elimination of item 5 altogether.

**Table 5:** Summary of the item discrimination index based on *n* = 136 physicians.

Subscale	Item discrimination index (scales based on Gerrity et al., 1995)^a^	Item discrimination index (scales based on Schneider et al., 2007)^b^	Item discrimination index (questionnaire without item 5)^c^
Anxiety due to uncertainty	0.61–0.83	0.59–0.82	0.59–0.82
Concern about bad outcomes	0.69–0.81	0.63–0.79	0.69–0.81
Reluctance to disclose uncertainty to patients	0.66–0.81	0.66–0.81	0.66–0.81
Reluctance to disclose mistakes to physicians	0.67	0.67	0.67

a Item 5 assigned to subscale “anxiety due to uncertainty.”

b Item 5 assigned to subscale “concern about bad outcomes.”

c Item 5 removed.

### Factorial validity

Mardia’s test indicated that the assumption of multivariate normality was violated. Consequently, we used a Satorra-Bentler scaled test statistic to address these violations and obtain robust fit indices (Satorra and Bentler, 1994). We estimated the robust chi-squared test statistic (Satorra and Bentler, 2010), and calculated the corresponding robust RMSEA (Brosseau-Liard et al., 2012), robust TLI and CFI (Brosseau-Liard and Savalei, 2014). All tested models revealed a Heywood case related to item 14, characterized by a negative variance estimate and a standardized factor loading greater than 1. Given that Heywood cases can result in biased estimations of fit indices, we chose to set the negative variance to zero across all models (Nachtigall et al., 2003). The results of all confirmatory factor analyses are summarized in Table 6.

**Table 6:** Model parameters and fit indices; calculations based on *n* = 136 physicians.

Fit indices	Model 1 (based on Gerrity et al., 1995)^a^	Model 2 (based on Schneider et al., 2007)^b^	Model 3 (PRU without item 5)^c^
Degrees of freedom	85	85	72
Fit indices			
Scaled Chi-Square test statistic	142.496**	142.834**	108.094**
CMIN/df	1.676	1.68	1.501
Robust RMSEA (lower CI-upper CI)	0.074 (0.052–0.095)	0.074 (0.053–0.095)	0.064 (0.037–0.088)
SRMR	0.072	0.072	0.067
Robust TLI	0.932	0.932	0.952
Robust CFI	0.945	0.945	0.962
AIC	6022.977	6022.386	5614.475
BIC	6124.920	6124.329	5710.593

a Item 5 assigned to subscale “anxiety due to uncertainty.”

b Item 5 assigned to subscale “concern about bad outcomes.”

c Item 5 removed; CMIN/df ≤3.

Robust RMSEA (95% confidence interval) < 0.06; SRMR <0.08; robust TLI and robust CFI >0.95; ** *p* < 0.01. PRU= Physicians’ Reaction to Uncertainty Scale; SRMR=Standardized Root Mean Square Residual.

Model 1 did not demonstrate an acceptable fit. All indices, except for the CMIN/df statistics and SRMR, fell out of an acceptable range, with RMSEA scores greater than 0.06 and TLI and CFI scores lower than 0.95. However, all item factor loadings were significant (*p* < 0.05), with standardized estimates ranging from 0.66 to 0.9. Additionally, all factor correlations were significant (*p* < 0.05), with values ranging from 0.2 to 0.72.

Model 2 similarly failed to achieve an acceptable fit. As with Model 1, all indices were outside the permissible range, except for the CMIN/df statistics and SRMR. All item factor loadings were significant (*p* < 0.05), with standardized estimates ranging from 0.67 to 0.9, and significant factor correlations (*p* < 0.05) ranging from 0.21 to 0.72.

**Figure 1:**
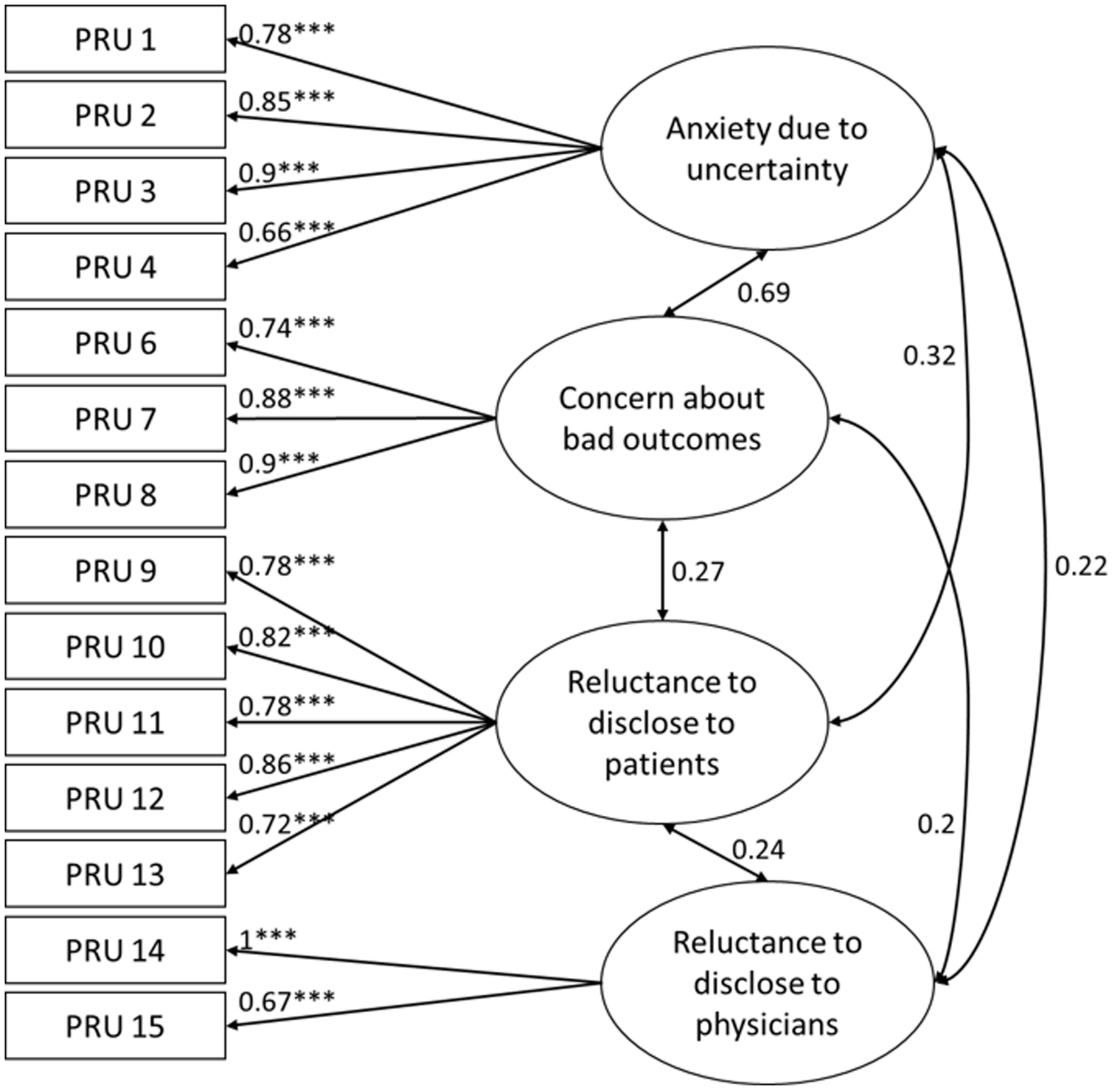
Model 3 showing the four factors of the Physicians’ Reaction to Uncertainty Scale with standardized coefficients of indicator variables and standardized covariances between factors; significance is only displayed for indicator variables with *** *p* < 0.001.

Model 3 (Figure 1) demonstrated an acceptable fit. The CMIN/df statistics, SRMR, robust TLI, and robust CFI scores met the defined cutoff criteria. Only the robust RMSEA score was slightly out of range. Again, all item factor loadings were significant (*p* < 0.05), with standardized estimates ranging from 0.66 to 0.9 and significant factor loadings (*p* < 0.05) between 0.2 and 0.69. Furthermore, the absolute AIC and BIC scores are lowest in Model 3, indicating a substantially better model fit compared to Model 1 and Model 2.

Due to the high factor correlation between “anxiety due to uncertainty” and “concern about bad outcomes,” we assessed discriminant validity following the study by Rönkkö and Cho (2022). The observed factor correlation of 0.69, with a confidence interval ranging from 0.57 to 0.81, indicated only marginal problems in discriminating between the two factors. The difference of the chi-square values—comparing a constrained model with the factor correlation fixed at 0.9 (χ^2^_0.9_ = 129.505) and the original model (χ^2^_org_ = 108.094)—also supported this conclusion, with χ^2^_0.9_ − χ^2^_org_ > 3.84.

## Discussion

This study aimed to psychometrically re-evaluate the German version of the Physician’s Reaction to Uncertainty scale among a heterogeneous group of physicians and examine its factorial structure through confirmatory factor analysis.

We successfully recruited 136 physicians for our survey, with the majority of them specializing in “general and internal medicine” and “pediatrics and adolescent medicine.” Participants reported a total of 22 different medical specialty backgrounds. Consequently, we achieved an important aspect of our first goal by recruiting a heterogeneous sample of physicians.

The reliability scores of the subscales were comparable to those found by Schneider et al. (2007). The assignment of item 5 to the subscale “anxiety due to uncertainty” or to the subscale “concern about bad outcomes” did not significantly impact the Cronbach’s alpha values. Nonetheless, the Cronbach’s alpha scores in our study were partly higher than those reported in the original version by Gerrity et al. (1995). Additionally, Schneider et al. (2007) reported higher Cronbach’s alpha values than those reported in a study by Gerrity et al. (1995), attributing this increase to their homogeneous sample and a more open attitude toward uncertainties and medical errors compared to the 1990s, when the original questionnaire was developed.

The item difficulty index showed acceptable values for the majority of items, except for item 14. The item discrimination index demonstrated very good values that are nearly similar to those of Schneider et al. (2007) for the subscales “anxiety due to uncertainty” and “concern about bad outcomes.” Notably, our sample exhibited higher values for the subscale “reluctance to disclose uncertainty to patients” and lower values for the subscale “reluctance to disclose mistakes to physicians.” The lower values for the latter are associated with the poor difficulty index of item 14 (“I almost never tell other physicians about diagnoses I have missed,” P*_14_* = 0.18), as 81.6% of participants selected one of the first response options. This response pattern substantially limits the variance, thereby reducing the discrimination index for this subscale. The extremely low difficulty of the item could reflect the importance of sharing uncertainties with colleagues, as reported by Han et al. (2021). At the same time, it contradicts a broad range of literature stating that physicians experience a variety of barriers to disclose medical errors, for example, due to social anxiety and the fear of being perceived as incompetent (Fattahi et al., 2025; Lipitz-Snyderman et al., 2017). At the same time, there is a consensus that open communication is an essential part of the medical profession, which might lead to a gap between knowing about the importance of disclosing uncertainties and actually doing it (Kaldjian, 2021). Considering the low difficulty of item 14, it may therefore reflect a social desirability bias rather than the use of a coping strategy.

The means and standard deviations of the majority of subscales were comparable to those reported by Schneider et al. (2007) but were lower than those reported by Gerrity et al. (1995). Given the heterogeneity of our sample, shifts in attitudes over the last 30 years may account for the differences observed when compared to Gerrity’s original scores. Indeed, the means of most subscales in our study are significantly lower than those reported by Gerrity et al. (1995), indicating reduced uncertainty scores in our sample. Supporting this interpretation, Simpkin and Armstrong (2019) noted that communicating uncertainties has become an integral part of shared decision-making (SDM) and is increasingly emphasized in guidelines and curricula. Härter et al. (2017) highlight several German projects and efforts aimed at implementing SDM. As a result, SDM cannot be considered without taking into account the aspect of uncertainty tolerance. Furthermore, a recent study demonstrated that greater uncertainty tolerance is associated with increased confidence in engaging in SDM (Valentine et al., 2024).

The confirmatory factor analysis revealed a Heywood case in our data, resulting in “improper solutions” of factor analyses (Van Driel, 1978). In our view, the lack of variance in item 14 contributed to this issue, along with the fact that the factor “reluctance to disclose mistakes to physicians” was represented by only two items. Typically, at least three items per factor are needed to prevent Heywood cases (Farooq, 2022). Since our goal was to validate the culturally adapted German version by Schneider et al. (2007), we refrained from developing new items. However, considering the methodological limitations of only two items representing a single factor, the instrument would benefit from incorporating additional items. As discussed above, the factor is highly relevant in terms of content and represents a central aspect of uncertainty. Therefore, we have opted not to exclude it from the analysis entirely. Since we could not resolve the underlying causes of the Heywood case, we set the negative variance to zero in all models (Nachtigall et al., 2003). While this is an established way for addressing issues arising from Heywood cases, it introduces methodological concerns, as in this case, the variance of the factor “reluctance to disclose mistakes to physicians” becomes solely dependent on the variance of item 15. Consequently, this reduces the overall reliability of the construct and limits the interpretability of the results. Nevertheless, the significant content relevance of this factor was deemed important enough to retain within the analysis.

Furthermore, the assignment of item 5 (“The uncertainty of patient care often troubles me.”) to a factor remained ambiguous, as indicated by the lower model fits of the first two models. This ambiguity may stem from the translation of the original term “troubles me” into the German term “Sorgen machen,” which translates to “worry.” On the one hand, “worrying” implies an attempt to control future events (Hoyer and Heidrich, 2009), aligning it more closely with the subscale “concern about bad outcomes.” In contrast, worrying is also closely related to anxiety (Borkovec et al., 1983), which places it nearer to the first subscale. Due to this ambiguity and supported by the AIC and BIC scores, we decided using a data-driven approach (Model 3) and eliminated item 5. This adjustment led to satisfactory model fits and may offer a better solution for the German version of the PRU. However, we were unable to confirm the factorial structure as proposed by Schneider et al. (2007). Further studies are necessary to validate the 14-item version.

The ambiguous assignment of item 5 also indicates the high correlation between the factors “concern about bad outcomes” and “anxiety due to uncertainty,” which we observed across all models. This issue was already described in the original study by Gerrity et al. (1995). Despite the high factor correlations, Gerrity et al. (1995) concluded that, although highly overlapping, the scales are sufficiently distinct to justify the use of two factors. Our findings are consistent with this conclusion. The examination of discriminant validity revealed only marginal problems, suggesting that it is “probably safe” to interpret both scales as separate constructs (Rönkkö and Cho, 2022, p. 36). This result supports the independent use of the PRU subscales.

Overall, based on our analyses, the 14-item German version of the PRU appears to be a reliable and valid questionnaire for assessing physicians’ responses to uncertainty, regardless of their medical specialty. This represents an important advantage, as the challenges arising from low uncertainty tolerance impact all types of physicians. In addition to personal consequences, such as increased burnout rates or reduced job satisfaction (Hancock and Mattick, 2020), a low level of uncertainty tolerance among physicians may also pose a burden on the entire healthcare system. Although findings are inconsistent, Strout et al. (2018) reported that lower uncertainty tolerance is associated with higher referral rates or perceived malpractice risk. Interventions to support physicians in better uncertainty tolerance are an important next step; the PRU appears to be a suitable tool for evaluating the effects of such interventions.

### Limitations

This study has some limitations. The online nature of our survey limits the generalizability of our findings. Despite our efforts to recruit physicians through various ways, we must assume a selection bias. Participants were required to possess at least a minimal level of confidence in using the internet, and they had to be a member of one of the aforementioned organizations. We also cannot rule out the possibility that a single person filled out the questionnaire multiple times. Furthermore, many physicians may find it uncomfortable to reveal their uncertainties, which could result in a self-selection bias favoring those who feel more confident in disclosing them. Increased confidence in disclosing uncertainties might reflect a higher uncertainty tolerance, which in turn could result in lower uncertainty scores. Additionally, our sample size may be considered insufficient, as rules of thumb suggest minimum sample sizes of 200 participants for confirmatory factor analyses (Jackson et al., 2013). Despite this, Tinsley and Tinsley (1987) recommend a minimum sample size of 5 to 10 participants per variable, while Ding et al. (1995) argue for a minimum sample size of 100 participants. Nevertheless, due to our limited sample size, our analyses may lack sufficient statistical power and need to be interpreted with caution. The fit indices might overestimate the true model fit, and parameters such as factor loadings and error variances could be inaccurate (Kyriazos, 2018; Wolf et al., 2013). The Heywood case highlights methodological limitations of the factor “reluctance to disclose mistakes to physicians,” which could be addressed by adding more items to better assess this construct. However, they provide valuable insights for evaluating the model structure of the German version of the PRU and contribute to further improving the instrument.

## Conclusion

The Physicians’ Reaction to Uncertainty Scale is a widely accepted questionnaire. Its international dissemination allows for the comparison of results across multiple studies. Furthermore, the subscales of the PRU enable the differentiation of different aspects of uncertainty. The removal of item 5 significantly improved the psychometric properties of the German version. The 14-item German version of the PRU demonstrates very good psychometric parameters not only for general practitioners but also for a heterogeneous sample of various specialties, making it a suitable instrument for studies on uncertainty. Future research should investigate the psychometric properties of the 14-item version in a larger sample of physicians.

## Data Availability

The raw data supporting the conclusions of this article will be made available by the authors, without undue reservation.

## References

[ref1] Akaike H. (1974). A new look at the statistical model identification. IEEE Trans. Autom. Control 19, 716–723. doi: 10.1109/TAC.1974.1100705

[ref2] Begin A. S. Hidrue M. Lehrhoff S. del Carmen M. G. Armstrong K. Wasfy J. H. (2022). Factors associated with physician tolerance of uncertainty: an observational study. J. Gen. Intern. Med. 37, 1415–1421. doi: 10.1007/s11606-021-06776-8, PMID: 33904030 PMC8074695

[ref3] Bland J. M. Altman D. G. (1997). Statistics notes: Cronbach’s alpha. BMJ 314:572. doi: 10.1136/bmj.314.7080.572, PMID: 9055718 PMC2126061

[ref4] Borkovec T. D. Robinson E. Pruzinsky T. DePree J. A. (1983). Preliminary exploration of worry: some characteristics and processes. Behav. Res. Ther. 21, 9–16. doi: 10.1016/0005-7967(83)90121-3, PMID: 6830571

[ref5] Bovier P. A. Perneger T. V. (2007). Stress from uncertainty from graduation to retirement—a population-based study of Swiss physicians. J. Gen. Intern. Med. 22, 632–638. doi: 10.1007/s11606-007-0159-7, PMID: 17443371 PMC1855273

[ref6] Brosseau-Liard P. E. Savalei V. (2014). Adjusting incremental fit indices for nonnormality. Multivar. Behav. Res. 49, 460–470. doi: 10.1080/00273171.2014.933697, PMID: 26732359

[ref7] Brosseau-Liard P. E. Savalei V. Li L. (2012). An investigation of the sample performance of two nonnormality corrections for RMSEA. Multivar. Behav. Res. 47, 904–930. doi: 10.1080/00273171.2012.715252, PMID: 26735008

[ref8] Burnham K. P. Anderson D. R. (Eds.). (2002). Model selection and multimodel inference: a practical information-theoretic approach. New York, NY: Springer New York.

[ref9] Carleton R. N. Norton M. A. P. J. Asmundson G. J. G. (2007). Fearing the unknown: a short version of the intolerance of uncertainty scale. J. Anxiety Disord. 21, 105–117. doi: 10.1016/j.janxdis.2006.03.014 16647833

[ref10] Chu E. C.-P. Yip A. S.-L. (2019). A rare presentation of benign acute childhood myositis. Clin. Case Rep. 7, 461–464. doi: 10.1002/ccr3.2001, PMID: 30899472 PMC6406135

[ref11] Cronbach L. J. (1951). Coefficient alpha and the internal structure of tests. Psychometrika 16, 297–334. doi: 10.1007/BF02310555

[ref12] Diedenhofen B. Musch J. (2016). Cocron: a web interface and R package for the statistical comparison of Cronbach’s alpha coefficients. Int. J. Internet Sci. 11, 51–60.

[ref13] Ding L. Velicer W. F. Harlow L. L. (1995). Effects of estimation methods, number of indicators per factor, and improper solutions on structural equation modeling fit indices. Struct. Equ. Model. Multidiscip. J. 2, 119–143. doi: 10.1080/10705519509540000

[ref14] Döring N. Bortz J. (2016). Forschungsmethoden und Evaluation in den Sozial- und Humanwissenschaften, Springer-Lehrbuch. Berlin, Heidelberg: Springer.

[ref15] Farooq R. (2022). Heywood cases: possible causes and solutions. Int. J. Data Anal. Tech. Strateg. 14:79. doi: 10.1504/IJDATS.2022.121506

[ref16] Fattahi H. Seproo F. G. Fattahi A. Rostami V. Shokri A. (2025). General practitioners’ perspectives on barriers to communication with specialists in the referral system: a systematic review and Meta-synthesis. Health Sci. Rep. 8:e70785. doi: 10.1002/hsr2.70785, PMID: 40415981 PMC12098960

[ref17] Gerrity M. S. White K. P. DeVellis R. F. Dittus R. S. (1995). Physicians’ reactions to uncertainty: refining the constructs and scales. Motiv. Emot. 19, 175–191. doi: 10.1007/BF02250510

[ref18] Han P. K. J. Klein W. M. P. Arora N. K. (2011). Varieties of uncertainty in health care: a conceptual taxonomy. Med. Decis. Mak. 31, 828–838. doi: 10.1177/0272989X10393976, PMID: 22067431 PMC3146626

[ref19] Han P. K. J. Strout T. D. Gutheil C. Germann C. King B. Ofstad E.. (2021). How physicians manage medical uncertainty: a qualitative study and conceptual taxonomy. Med. Decis. Mak. 41, 275–291. doi: 10.1177/0272989X21992340, PMID: 33588616 PMC7985858

[ref20] Hancock J. Mattick K. (2020). Tolerance of ambiguity and psychological well-being in medical training: a systematic review. Med. Educ. 54, 125–137. doi: 10.1111/medu.14031, PMID: 31867801 PMC7003828

[ref21] Härter M. Dirmaier J. Scholl I. Donner-Banzhoff N. Dierks M.-L. Eich W.. (2017). The long way of implementing patient-centered care and shared decision making in Germany. Z. Evid. Fortbild. Qual. Gesundhswes. 123–124, 46–51. doi: 10.1016/j.zefq.2017.05.006 28546055

[ref22] Hillen M. A. Gutheil C. M. Strout T. D. Smets E. M. A. Han P. K. J. (2017). Tolerance of uncertainty: conceptual analysis, integrative model, and implications for healthcare. Soc. Sci. Med. 180, 62–75. doi: 10.1016/j.socscimed.2017.03.024, PMID: 28324792

[ref23] Hoyer J. Heidrich S. (2009). Wann Sind Sorgen pathologisch? Verhaltenstherapie 19, 33–39. doi: 10.1159/000201938

[ref24] Hu L. Bentler P. M. (1999). Cutoff criteria for fit indexes in covariance structure analysis: conventional criteria versus new alternatives. Struct. Equ. Model. Multidiscip. J. 6, 1–55. doi: 10.1080/10705519909540118

[ref25] Jackson D. L. Voth J. Frey M. P. (2013). A note on sample size and solution propriety for confirmatory factor analytic models. Struct. Equ. Model. Multidiscip. J. 20, 86–97. doi: 10.1080/10705511.2013.742388

[ref26] Kaldjian L. C. (2021). Communication about medical errors. Patient Educ. Couns. 104, 989–993. doi: 10.1016/j.pec.2020.11.035, PMID: 33280965

[ref27] Kline R. B. (1998). Principles and practice of structural equation modeling. New York, NY: Guilford Press.

[ref28] Korkmaz S. Goksuluk D. Zararsiz G. (2014). MVN: an R package for assessing multivariate normality. The R Journal. vol. 6, 151–162.

[ref29] Kyriazos T. A. (2018). Applied psychometrics: sample size and sample power considerations in factor analysis (EFA, CFA) and SEM in general. Psychology 9, 2207–2230. doi: 10.4236/psych.2018.98126

[ref30] Lipitz-Snyderman A. Kale M. Robbins L. Pfister D. Fortier E. Pocus V.. (2017). Peers without fears? Barriers to effective communication among primary care physicians and oncologists about diagnostic delays in cancer. BMJ Qual. Saf. 26, 892–898. doi: 10.1136/bmjqs-2016-006181, PMID: 28655713 PMC5953211

[ref31] Mardia K. V. (1974). Applications of some measures of multivariate skewness and kurtosis in testing normality and robustness studies. Sankhyā Indian J. Stat. Ser. B (1960-2002). 36, 115–128. Available at: http://www.jstor.org/stable/25051892

[ref32] Nachtigall C. Kroehne U. Funke F. Steyer R. (2003). Pros and cons of structural equation modeling. Methods Psychol. Res. Online 8, 1–22. doi: 10.23668/psycharchives.12783

[ref33] R Core Team. (2023). R: A language and environment for statistical computing. R Foundation for Statistical Computing, Vienna, Austria.

[ref34] Raftery A. E. (1995). Bayesian model selection in social research. Sociol. Methodol. 25, 111–163. doi: 10.2307/271063

[ref35] Rönkkö M. Cho E. (2022). An updated guideline for assessing discriminant validity. Organ. Res. Methods 25, 6–14. doi: 10.1177/1094428120968614

[ref36] Rosseel Y. (2012). Lavaan: an R package for structural equation modeling. J. Stat. Softw. 48, 1–36. doi: 10.18637/jss.v048.i02

[ref37] Satorra A. Bentler P. M. (1994). “Corrections to test statistics and standard errors in covariance structure analysis,” in Latent variables analysis: Applications for developmental research Sage Publications, Inc. eds. A. von Eye and C. C. Clogg. 399–419.

[ref38] Satorra A. Bentler P. M. (2010). Ensuring Positiveness of the scaled difference chi-square test statistic. Psychometrika 75, 243–248. doi: 10.1007/s11336-009-9135-y, PMID: 20640194 PMC2905175

[ref39] Schneider A. Szecsenyi J. Barie S. Joest K. Rosemann T. (2007). Validation and cultural adaptation of a German version of the physicians’ reactions to uncertainty scales. BMC Health Serv. Res. 7, 1–6. doi: 10.1186/1472-6963-7-81 17562018 PMC1903353

[ref40] Schwarz G. (1978). Estimating the dimension of a model. Ann. Stat. 6, 461–464. doi: 10.1214/aos/1176344136

[ref41] Scott I. A. Doust J. A. Keijzers G. B. Wallis K. A. (2023). Coping with uncertainty in clinical practice: a narrative review. Med. J. Aust. 218, 418–425. doi: 10.5694/mja2.51925, PMID: 37087692

[ref42] Seoni S. Jahmunah V. Salvi M. Barua P. D. Molinari F. Acharya U. R. (2023). Application of uncertainty quantification to artificial intelligence in healthcare: a review of last decade (2013–2023). Comput. Biol. Med. 165:107441. doi: 10.1016/j.compbiomed.2023.107441, PMID: 37683529

[ref43] Simpkin A. L. Armstrong K. A. (2019). Communicating uncertainty: a narrative review and framework for future research. J. Gen. Intern. Med. 34, 2586–2591. doi: 10.1007/s11606-019-04860-8, PMID: 31197729 PMC6848305

[ref44] Stephens G. C. Lazarus M. D. Sarkar M. Karim M. N. Wilson A. B. (2023). Identifying validity evidence for uncertainty tolerance scales: a systematic review. Med. Educ. 57, 844–856. doi: 10.1111/medu.15014 36576391

[ref45] Streiner D. L. Kottner J. (2014). Recommendations for reporting the results of studies of instrument and scale development and testing. J. Adv. Nurs. 70, 1970–1979. doi: 10.1111/jan.12402, PMID: 24684713

[ref46] Strout T. D. Hillen M. Gutheil C. Anderson E. Hutchinson R. Ward H.. (2018). Tolerance of uncertainty: a systematic review of health and healthcare-related outcomes. Patient Educ. Couns. 101, 1518–1537. doi: 10.1016/j.pec.2018.03.030, PMID: 29655876

[ref47] Tinsley H. E. A. Tinsley D. J. (1987). Uses of factor analysis in counseling psychology research. J. Couns. Psychol. 34, 414–424. doi: 10.1037/0022-0167.34.4.414

[ref48] Valentine K. Leavitt L. Sepucha K. R. Atlas S. J. Simmons L. Siegel L.. (2024). Uncertainty tolerance among primary care physicians: relationship to shared decision making-related perceptions, practices, and physician characteristics. Patient Educ. Couns. 123:108232. doi: 10.1016/j.pec.2024.108232, PMID: 38458091 PMC10997439

[ref49] Van Driel O. P. (1978). On various causes of improper solutions in maximum likelihood factor analysis. Psychometrika 43, 225–243. doi: 10.1007/BF02293865

[ref50] Wolf E. J. Harrington K. M. Clark S. L. Miller M. W. (2013). Sample size requirements for structural equation models: an evaluation of power, bias, and solution propriety. Educ. Psychol. Meas. 73, 913–934. doi: 10.1177/0013164413495237, PMID: 25705052 PMC4334479

