# Supplementary material for: Psychometric re-evaluation of the German version of the Physicians’ Reaction to Uncertainty Scale
Source: Front Psychol. 2025 Aug 6;16:1552177. doi: 10.3389/fpsyg.2025.1552177 (PMC12365602; doi:10.3389/fpsyg.2025.1552177)
Supplement: Supplementary file 1 [file Supplementary_file_1.docx]

**Additional File 1**

*Comprehensive list of medical specialties*

| Medical specialties | n |
| --- | --- |
| Ophthalmology | 2 |
| (Neuro)surgery | 14 |
| Gynaecology | 4 |
| Gastroenterology | 4 |
| General and internal medicine | 45 |
| Dermatology | 3 |
| Ear, nose and throat medicine | 1 |
| Human genetics | 2 |
| Cardiology | 1 |
| Pediatrics and adolescent medicine | 42 |
| Pulmonology | 1 |
| Neurology | 6 |
| Nephrology | 1 |
| Oncology | 1 |
| Orthopaedics | 2 |
| Pathology | 1 |
| Psychiatry | 1 |
| Psychosomatics | 3 |
| Rheumatology | 1 |
| Pain therapy | 1 |
| Environmental medicine | 1 |
| Other medical specialty not mentioned | 14 |
