# Supplementary material for: Psychometric re-evaluation of the German version of the Physicians’ Reaction to Uncertainty Scale
Source: Front Psychol. 2025 Aug 6;16:1552177. doi: 10.3389/fpsyg.2025.1552177 (PMC12365602; doi:10.3389/fpsyg.2025.1552177)
Supplement: Supplementary file 2 [file Supplementary_file_2.docx]

**Additional File 2**

*German version of the Physicians’ Reaction to Uncertainty Scale (PRU) (Schneider et al.,2007)*

| Subscale | Item |
| --- | --- |
| Anxiety due to uncertainty | 1) In der Regel bin ich beunruhigt, wenn ich mir bei einer Diagnose nicht sicher bin. |
|  | 2) Ich finde die mit der Behandlung von Patient:innen verbundene Unsicherheit irritierend. |
|  | 3) Unsicherheit in der Patientenbehandlung beunruhigt mich. |
|  | 4) Ich kann die Unsicherheit in der Behandlung gut tolerieren.* |
| Concern about bad outcomes | 5) Die Unsicherheit bei der Patientenbehandlung macht mir oft Sorgen. |
|  | 6) Wenn ich mir bei einer Diagnose unsicher bin, stelle ich mir alle Arten schlimmer Szenarien vor – der/die Patient:in stirbt, der/die Patient:in verklagt mich, etc. |
|  | 7) Ich habe Angst, für mein begrenztes Wissen verantwortlich gemacht zu werden. |
|  | 8) Ich habe Angst vor einem Kunstfehler, wenn ich die Diagnose nicht kenne. |
| Reluctance to disclose uncertainty to patients | 9) Wenn Ärzt:innen bei einer Diagnose unsicher sind, sollten sie dies den Patient:innen mitteilen.* |
|  | 10) Ich teile meinen Patient:innen meine Unsicherheit immer mit.* |
|  | 11) Wenn ich alle meine Unsicherheiten meinen Patient:innen mitteilen würde, würden sie ihr Vertrauen in mich verlieren. |
|  | 12) Das Mitteilen meiner Unsicherheit verbessert die Beziehung zu meinen Patient:innen.* |
|  | 13) Ich ziehe es vor, wenn Patient:innen nicht wissen, dass ich mir unsicher bin, welche Behandlung ich wählen soll. |
| Reluctance to disclose mistakes to physicians | 14) Ich erzähle anderen Ärzt:innen fast nie von Diagnosen, die ich übersehen habe. |
|  | 15) Ich erzähle anderen Ärzt:innen nie von Behandlungsfehlern, die ich gemacht habe. |
|  |  |

Note. Rating of items on a 6-point Likert scale ranging from “ich widerspreche sehr” to “ich stimme sehr zu”; * items are inversely coded
